# Conservatism and “copy-if-better” in chimpanzees (*Pan troglodytes*)

**DOI:** 10.1007/s10071-016-1061-7

**Published:** 2016-12-20

**Authors:** Edwin J. C. van Leeuwen, Josep Call

**Affiliations:** 1 School of Psychology and Neuroscience, University of St Andrews, St Mary’s Quad, South Street, St Andrews, KY16 9JP UK; 2 Max Planck Institute for Psycholinguistics, Wundtlaan 1, 6525 XD Nijmegen, The Netherlands; 3 Max Planck Institute for Evolutionary Anthropology, Deutscher Platz 6, 04103 Leipzig, Germany

**Keywords:** Culture, Social learning, Chimpanzees, Decision-making

## Abstract

Social learning is predicted to evolve in socially living animals provided the learning process is not random but biased by certain socio-ecological factors. One bias of particular interest for the emergence of (cumulative) culture is the tendency to forgo personal behaviour in favour of relatively better variants observed in others, also known as the “copy-if-better” strategy. We investigated whether chimpanzees employ copy-if-better in a simple token-exchange paradigm controlling for individual and random social learning. After being trained on one token-type, subjects were confronted with a conspecific demonstrator who either received the same food reward as the subject (control condition) or a higher value food reward than the subject (test condition) for exchanging another token-type. In general, the chimpanzees persisted in exchanging the token-type they were trained on individually, indicating a form of conservatism consistent with previous studies. However, the chimpanzees were more inclined to copy the demonstrator in the test compared to the control condition, indicating a tendency to employ a copy-if-better strategy. We discuss the validity of our results by considering alternative explanations and relate our findings to the emergence of cumulative culture.

**Electronic supplementary material:**

The online version of this article (doi:10.1007/s10071-016-1061-7) contains supplementary material, which is available to authorized users.

## Introduction

Social learning, the form of learning that is influenced by observation of, or interaction with, another animal or its products (Heyes 1994), is expected to evolve in social animals as an alternative to relatively time-consuming and risky individual exploration tendencies (Danchin et al. 2004). In fact, social learning is the preferred mode of information acquisition when individual learning is costly—however, social learning needs to be guided by certain socio-ecological factors to become adaptive (Boyd and Richerson 1985). These factors have been referred to as “social learning strategies” (Laland 2004). One such social learning strategy concerns the relative pay-offs across conspecifics: if an animal observes a conspecific using a different yet more efficient behavioural variant, this animal could be expected to abandon its behaviour in favour of the observed one (Laland 2004). Such a “copy-if-better” strategy is particularly relevant to the emergence of cumulative culture, the incremental ratcheting of socially acquired information (Tennie et al. 2009). Human societies are prime examples of cumulative culture, arguably lacking an equivalent in other species, including our closest living relative the chimpanzee (Mesoudi 2011), despite their cultural nature (e.g. Luncz et al. 2012; van Leeuwen et al. 2012). Here, we focus on a question that could shed light on the seeming absence of cumulative culture in chimpanzees: do chimpanzees copy a conspecific using a more rewarding behavioural variant?

Several studies have shown that chimpanzees are conservative when it comes to abandoning learned behaviours (e.g. Hrubesch et al. 2009; Marshall-Pescini and Whiten 2008). Although chimpanzees can be flexible when changing behaviour yields more rewards (van Leeuwen et al. 2013; Yamamoto et al. 2013), confounding factors like variant preferences (i.e. one behavioural variant e.g. “poking” is intrinsically preferred over another behavioural variant e.g. “lifting”, see Hopper et al. 2007) and individual learning (i.e. despite social information being available, subjects make use of their own trial and error sampling efforts) may have been responsible for these results. Thus, it is currently unknown whether chimpanzees engage in copy-if-better based purely on social information. Here, we investigated whether chimpanzees copied better behavioural variants from a conspecific demonstrator using a token-exchange paradigm which allowed us to control for variant preferences and individual learning. Moreover, by incorporating a control condition in which the conspecific demonstrator used another token-type for the *same* reward as the subject, we were additionally able to account for social learning tendencies other than the copy-if-better strategy. We hypothesized that chimpanzees would predominantly rely on their individually learned behaviour (sensu Hrubesch et al. 2009; Marshall-Pescini and Whiten 2008), but also be more inclined to copy their conspecific when she received a relatively high value reward (test condition) compared to when both chimpanzees received equal rewards (control condition).

## Materials and methods

**Fig. 1:**
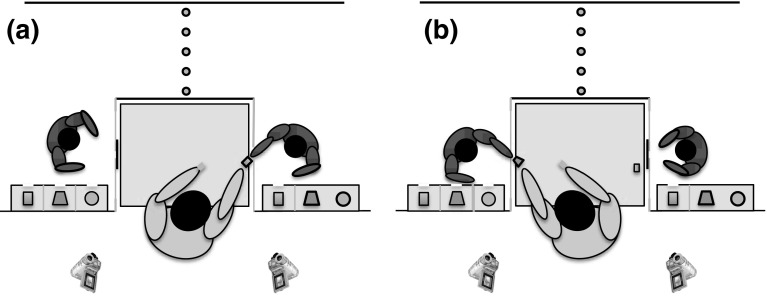
Depicted are **a** the demonstrator exchanging a rectangle-shaped token with the experimenter for a piece of carrot, and subsequently **b** the subject exchanging a *trapezium-shaped* token with the experimenter for a piece of carrot. The subject (on the *left* in both figures) had a private token container available, comprising three compartments holding ~40 tokens of the depicted token-type each. The demonstrator had the same token container available, yet was only able to obtain one particular token-type (here: *rectangle-shaped*). In order to make the demonstrator’s exchanges and associated food rewards conspicuous to the subject, the experimenter held the exchanged token and the food reward in the air for ~2 s after which he gave the reward to the demonstrator and placed the exchanged token on the table (largest *grey rectangle*). After any exchange, the experimenter would block the used exchange hole with a piece of perspex (*black vertical line*) and orient his body towards the other chimpanzee in anticipation of a new exchange. In the test condition, the demonstrator received one piece of banana instead of carrot per exchanged token. All food rewards were hidden underneath the table, out of sight of both chimpanzees

We tested 12 chimpanzees at the Wolfgang Kohler Primate Research Center in Leipzig, Germany (6 males; *M*_age_ = 20.1 years, range = 6.4–40.0 years). One of these chimpanzees acted as demonstrator throughout the entire study (Sandra, female, 22.5 years). First, subjects were individually trained to exchange tokens of one particular token-type with the experimenter for one piece of carrot (low value reward) per token for two sessions on two consecutive days (10 trials per session). During training, the subjects did not experience the existence of the other two token-types (i.e. we used three different token-types throughout the entire study). Second, within 3 days of completing training, the subjects were paired with the demonstrator for testing, i.e. the subject and demonstrator were invited in adjacent rooms—without having access to, but with clear visibility of each other’s rooms—and the experimenter would alternate between facilitating an exchange with the subject and demonstrator (Fig. 1).

Testing consisted of a control (equal food rewards: both carrot) and a test condition (higher value food reward for the demonstrator: banana vs. carrot), administered in an a priori counterbalanced order. Each condition comprised of two sessions, administered on two consecutive days. Per session, the experimenter would alternate between exchanging one reward per token with the subject and the demonstrator for a total of 10 trials each, whereby the subject was afforded the first exchange and the demonstrator would always exchange another token-type than the subject was trained on. To prevent carry-over effects for the subject, the demonstrator used a different token-type per condition. A total of 360 subject trials were administered. Importantly, we chose to *not* reward the subject with banana whenever the subject switched to using the demonstrator token in the test condition for the reason that all its subsequent trials would be influenced by this individual learning experience. Instead, we always rewarded the subject with carrot. Accordingly, in addition to analysing subjects’ full set of trials, we also analysed subjects’ *first* responses after having observed the demonstrator. Whereas this first decision after observing the demonstrator is arguably the cleanest test of the copy-if-better strategy (i.e. the observer has not yet personally experienced the contingencies of the different token-types), the analysis of the full set of trials remains relevant for it may take subjects, especially conservative ones like chimpanzees (e.g. see Hrubesch et al. 2009; Marshall-Pescini and Whiten 2008), repeated exposure to better variants before they would attempt to copy them.

First, we investigated whether the chimpanzees selectively chose between the three token-types with permutation tests (*n* = 1000; script available upon request). Second, we used two GLMMs with binomial error structure and logit link function (with response variable “yes/no used trained token” and “yes/no copied the demonstrator”, respectively) to investigate whether the chimpanzees made different choices in the control versus test condition. In both models, we controlled for the “token-type the subject was trained on”, the “token-type used by the stooge”, and the “order” of conditions as fixed effects, and “subject”, “test-date” and “trials within subject” as random effects. Our variable of interest was “condition”, which we thus added as fixed effect. We also added the variable “trial” as fixed effect, reflecting a time component (20 trials per condition). Since we expected that the effect of “condition” on the chimpanzees’ responses could be moderated by time, we included the interaction between condition and trial in both models. Furthermore, to account for the times the subject observed the demonstrator exchanging, we included “observed stooge-trials” (*ad hoc* assessed during the experiment by the experimenter) as an offset term in our models. Finally, in the second model (with response variable “yes/no copied the demonstrator”), we excluded the first trial for each subject per condition, because prior to this first trial the subjects had not observed the demonstrator exchanging tokens yet.

Our GLMM tests commenced with full–null model comparisons, where the full model comprised all terms described above, and the null model comprised all terms except for the terms “condition” and “trial”. Only upon finding a significant full–null model comparison (*p* < 0.05) would we proceed with exploring the effects within the full model. Note that in both models “subject” was specified as a random effect for we aimed to make general inferences about the behaviour of chimpanzees, not about the particular chimpanzees studied. Nevertheless, we report individual decisions in Supplementary Table S1. Relatedly, we did not search for any model biases for we anticipated the copy-if-better strategy to supersede any preferences for *whom* to copy. For more details on the experimental procedure and analysis, see ESM.

## Results

### Are chimpanzees conservative?

**Fig. 2:**
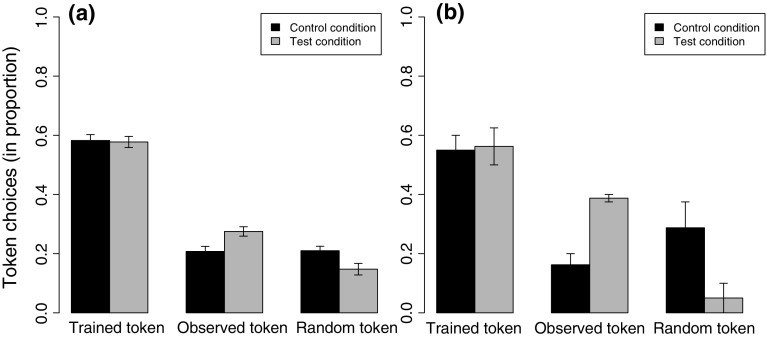
Subjects’ token choices (mean proportion ± s.e.m) for **a** all data, **b** the first trial after having observed the demonstrator receiving either the same reward as the subject (control condition) or a higher value reward (test condition) for using a different token-type

The chimpanzees mainly exchanged the token-type they had been trained on (Permutation test across conditions: *χ* ^2^ = 151.3, *p* < 0.001; Fig. 2a), both in the control (*χ* ^2^ = 76.0, *p* < 0.001) and test condition (*χ* ^2^ = 78.7, *p* < 0.001). Given that the full–null model comparison was non-significant (GLMM, LRT: *χ* ^2^ = 4.20, Δdf = 3, *p* = 0.24), there was little evidence of a difference in the probability a chimpanzee would exchange its trained token-type between conditions or across time.

### Do chimpanzees copy-if-better?

When chimpanzees did not choose their trained token-type (approx. 40% of the exchanges), they preferred to exchange the demonstrator’s token over the third option, but only when the demonstrator received banana for her token (test condition: *χ* ^2^ = 8.07, *p* = 0.017; control condition *χ* ^2^ = 0.02, *p* = 1; Fig. 2a). This result was corroborated by the trend in the data set only comprising subjects’ *first* trials after observing the demonstrator (test condition: *χ* ^2^ = 5.79, *p* = 0.062; control condition: *χ* ^2^ = 0.64, *p* = 0.74; Fig. 2b). A significant full–null GLMM model comparison (*χ* ^2^ = 14.70, Δd*f* = 3, *p* < 0.003) allowed us to inspect chimpanzees’ responses in more detail. There was little evidence that chimpanzees were affected by time differently between the two conditions (interaction trial and condition: *χ* ^2^ = 1.54, Δd*f* = 1, *p* = 0.21). However, chimpanzees were more likely to copy the demonstrator when she received banana versus carrot (main effect for condition: *χ* ^2^ = 9.17, Δd*f* = 1, *p* < 0.003; Estimate ± SE = −2.13 ± 0.80). This result was robust against sequential single-trial omissions (range of the condition estimate: −1.95 to −2.25). For more details on the results, see ESM.

## Discussion

Chimpanzees were predominantly conservative as they remained faithful to their trained token-type, both in the control and test condition. Yet, some chimpanzees changed their behaviour, and when this was the case (~40% of the trials), they copied the demonstrator more readily when the demonstrator used a *better* behavioural variant compared to when the demonstrator used an equally profitable variant as the subject. Taken together, our results suggest that chimpanzees were mostly conservative, but that at least some chimpanzees are also capable of applying a copy-if-better strategy.

Identifying changes in chimpanzees’ preferences is important because it suggests that chimpanzees’ conservatism (e.g. Hrubesch et al. 2009; Marshall-Pescini and Whiten 2008) is neither unconditional nor unaffected by purely social information (this study). Moreover, abandoning familiar behaviour in favour of a more profitable strategy induced by observational learning would allow for the ratcheting effect crucial to cumulative culture (Tennie et al. 2009).

Two previous studies have explicitly documented switching behaviour in chimpanzees when social information was available (see van Leeuwen et al. 2013; Yamamoto et al. 2013). These studies thus provided evidence for chimpanzees being able to overcome their seemingly potent conservatism (e.g. Hrubesch et al. 2009; Marshall-Pescini and Whiten 2008). However, in both studies, mechanisms other than copy-if-better could have explained the observed switching patterns. For instance, despite social information being available, in both studies, the chimpanzees could have relied solely on their individual learning tendencies, especially given that the socially demonstrated variant was also more rewarding (see van Leeuwen et al. 2013; Yamamoto et al. 2013). This is a plausible alternative explanation because chimpanzees are known to occasionally explore different solutions regardless of social information (e.g. see Bonnie et al. 2012; Dean et al. 2012): If they happened to stumble upon a highly rewarding solution, they might switch their preference in line with optimal foraging behaviour instead of based on social information. Similarly, when the two experimentally induced variants differ qualitatively (i.e. the familiar variant and the demonstrated alternative), chimpanzees may simply switch to the alternative variant based on their intrinsic variant preference [i.e. in Yamamoto et al. (2013), the chimpanzees could have preferred to suck through a straw over dipping the straw]. Finally, chimpanzees (or social animals in general) may decide to use social information regardless of its associated benefit [e.g. see van Leeuwen et al. (2014); grass in ear]. If chimpanzees are only presented with social information associated with more profitable outcomes compared to their familiar behavioural variant [as in both van Leeuwen et al. (2013) and Yamamoto et al. (2013)], it remains impossible to disentangle mere social learning from the copy-if-better strategy. Note that this is the crux of the copy-if-better strategy: only when the observed variant yields more or better rewards than one’s own variant would one be inclined to adopt the observed variant (see Laland 2004).

Our design allowed us to control for the confounds that may have operated in previous studies (e.g. van Leeuwen et al. 2013; Yamamoto et al. 2013). First, by offering the chimpanzees three instead of two behavioural variants, we prevented the chimpanzees from coincidentally (rather than selectively) adopting the demonstrated variant when they, for some non-social reason (i.e. the mere presence of high value food), decided to forgo their familiar variant. The fact that the chimpanzees in our study chose the demonstrated variant significantly more often than the third option in the test condition (demonstrator received higher value reward) suggests that the chimpanzees selectively opted for the demonstrated variant instead of merely exploring other alternatives. Moreover, by not rewarding the subjects with the higher value food reward upon their copying of the demonstrator (in the test condition), we minimized the possibility that the chimpanzees acquired the demonstrated variant by mere individual sampling (and subsequent associative learning). Second, by only using qualitatively *equal* variants (i.e. all variants consisted of the same token-exchange sequence), we prevented chimpanzees from adopting the demonstrated variant because of intrinsic preferences (note that we additionally controlled for “token-type” in our statistical analysis). Finally, we precluded mere social learning as explanatory variable by incorporating a control condition in which subjects observed the demonstrator receiving an equally valuable food reward as themselves, which formed the benchmark for our investigation of chimpanzees’ copy-if-better tendencies (as operationalized in the test condition).

Despite our stringent procedure, we detected some indication of a copy-if-better strategy. By showing that the chimpanzees in our study were more inclined to adopt the demonstrated variant in the test versus control condition, and that within the test condition, the chimpanzees were more inclined to use the demonstrated compared to the random variant, we showed that some chimpanzees, despite their dominant tendency to remain conservative, applied a copy-if-better strategy. Hence, our study shows that the cognitive capacity underlying chimpanzees adopting better behavioural variants (e.g. van Leeuwen et al. 2013; Yamamoto et al. 2013) could actually be the copy-if-better strategy instead of individual or unbiased social learning. Subjects’ first choices after observing the demonstrator exchanging for the first time (Fig. 2b) are particularly informative here because they reflect subjects’ inclination to copy the demonstrator before personally experiencing its consequences. Future work should corroborate our findings by adopting a study design in which subjects experience such first choices repetitively (e.g. by using different sets of tokens). Moreover, it would be valuable to titrate chimpanzees’ proclivity to remain conservative in the face of increasingly beneficial alternative variants. Despite our study showing that a copy-if-better strategy is not an insurmountable obstacle for at least some chimpanzees, such additional research is needed to establish whether the extent to which chimpanzees use the copy-if-better strategy might be related to their relatively under-expressed forms of culture in comparison to humans (Mesoudi 2011).

## Electronic supplementary material

Below is the link to the electronic supplementary material.

Supplementary material 1 (PDF 203 kb)

Supplementary material 2 (PDF 40 kb)
