# Supplementary material for: Conservatism and “copy-if-better” in chimpanzees (Pan troglodytes)
Source: Anim Cogn. 2016 Dec 20;20(3):575–9. doi: 10.1007/s10071-016-1061-7 (PMC5394136; doi:10.1007/s10071-016-1061-7)
Supplement: Supplementary file 1 — Supplementary material 1 (PDF 203 kb) [file 10071_2016_1061_MOESM1_ESM.pdf]

Electronic Supplementary Material for

Conservatism and 'copy-if-better' in chimpanzees (*Pan troglodytes*)

Edwin J.C. van Leeuwen[1,2*], Josep Call[1,3]

[1] University of St Andrews, School of Psychology and Neuroscience, St Mary's Quad, South Street, KY16 9JP, St Andrews, United Kingdom

[2] Max Planck Institute for Psycholinguistics, Wundtlaan 1, Nijmegen, the Netherlands

[3] Max Planck Institute for Evolutionary Anthropology, Deutscher Platz 6, 04103 Leipzig, Germany

[*] Corresponding author (ejcvanleeuwen@gmail.com)

MATERIAL AND METHODS

Ethics statement

This study was approved by the Max Planck Institute for Evolutionary Anthropology (Leipzig). We certify that we followed the “Principles for the Ethical treatment of nonhuman primates” (IPS guidelines, 2007), that the research adhered to the ASAB/ABS Guidelines for the Use of Animals in Research, that all animal husbandry procedures were non-invasive and that participation by the chimpanzees was voluntary. Normal diets were not restricted; the chimpanzees gained extra food by participating. The chimpanzees lived in a social group of 17 individuals. They had access to an indoor (430 m^2) and outdoor enclosure (4,000 m^2). All enclosures include climbing structures, natural vegetation, and forms of enrichment (puzzle-boxes, jute bags, provisioning of concealed food). For the experiment, the chimpanzees were enticed into observation rooms, where they could be tested in two separate, adjacent rooms. A research booth in between the two observations rooms allowed the experimenter to conduct the study.

Experimental procedure

The chimpanzees in the Wolfgang Kohler Primate Research Center (Leipzig) prefer banana over carrot [1]. Due to logistical challenges, not all subjects completed their four test sessions, resulting in a total of 360 subject trials. One session of one chimpanzee was discarded entirely, because she sat underneath the exchange window with her back toward the experimenter and the demonstrator while exchanging tokens over her head (i.e. the subject could not possibly have observed the demonstrator exchanging).

Alternating exchanges between the subject and demonstrator was facilitated by the experimenter blocking the exchange location with a small piece of perspex right after the exchange. Subsequently, the experimenter would orient toward the other

chimpanzee, while ignoring possible exchange attempts by the chimpanzee who just exchanged a token with the experimenter (to prevent the building up of frustration, the experimenter attuned to the speed of the subject, which worked adequately due to the continued and predictable exchange behaviour of the demonstrator).

During testing, the subject and the demonstrator had all three token-types available *ad libitum*. The demonstrator, however, could only use one particular token-type due to access to the other token-types being blocked with transparent barricades. The subject was unable to see these barricades, resulting in the desired situation where the subject plausibly perceived the demonstrator making a free choice between the available token-types.

Throughout testing, the experimenter maximized the number of demonstrator-trials that the subject would observe by i) having all commodities concentrated in one small space (token containers, exchange windows, food rewards), ii) placing the exchanged tokens on a table connecting the exchange-windows of the subject and the demonstrator, iii) calling out the name of the subject during the demonstrator-exchange, and iv) timing the demonstrator-exchange such that the subject would likely observe it (i.e. most subjects remained close to the exchange window throughout the testing phases, yet some walked around in a somewhat predictable pattern). Nevertheless, not all demonstrator exchanges were observed by the subjects, so we included “observed demonstrator-trials” (*ad-hoc* assessed during the experiment by the experimenter) as an offset term in our models.

Notably, by counterbalancing the token-types that the subjects were trained on in relation to the token-types used by the demonstrator, we were able to control for the effect of potential token preferences (these factors were additionally controlled for in the models). Similarly, by using three different token-types, we were able to control for individual learning: in case subjects abandoned their trained token and started exploring

the other tokens, they may not have necessarily selected the demonstrator's token, which would have been the case if we had only used two different token-types.

Analysis

We opted for initial permutation tests because the token choices were non-independent, both within and across subjects, and the binomial Generalized Linear Mixed Models (GLMMs) could not ascertain whether certain token-types were chosen more than specific others due to the fact that we used three instead of two token-type alternatives (i.e. the GLMM intercept estimate would indicate whether token-type A was used more often than token-types B and C combined).

In the first GLMM, the response variable was "yes/no used trained token", in keeping with predictions based on previous research [2, 3]. In the second GLMM the response variable was "yes/no copied the demonstrator", in keeping with our 'copy-if-better' hypothesis (based on [4, 5]).

The GLMMs were fitted in R [6] using the function lmer of the R-package lme4 [7]. The significance of the full model as compared to the null model (comprising only the control variables, including the random effects) was established using a likelihood ratio test (R function anova with argument test set to "Chisq"; [8]). To allow for a likelihood ratio test we fitted the models using Maximum Likelihood (rather than Restricted Maximum Likelihood; [9]). P-values for the individual effects were based on likelihood ratio tests comparing the deviances of the full and respective reduced models.

RESULTS

Do chimpanzees copy if better?

Here, we present additional information regarding chimpanzees' proclivity to copy the demonstrator. First, chimpanzees' individual choices on their first trials after having observed the demonstrator are depicted in Table S1.

Table S1. Proportion of first trials (2 per condition, given that each condition comprised 2 sessions of 10 trials each) in which the demonstrator was copied. In the control condition, the demonstrator received a similar-value reward for exchanging as the subject (carrot), in the test condition the demonstrator received a higher-value reward for exchanging than the subject (banana). When the demonstrator was not copied (e.g. when the proportion is "0"), the subject either chose its trained token or the random one. Note that individual variation exists such that only some chimpanzees (n=4) copied-if-better on their *first* trials.

Chimpanzee	*Control condition*	*Test condition*
Bangolo	0	0
Dorien	n/a*	1
Frodo	0	n/a**
Kara	0	1
Kofi	0	0
Lobo	0	0
Lome	0	0
Riet	0	1
Robert	1	0
Svela	0	0
Tai	0.5	1

*no valid results were obtained due to subject not observing the demonstrations
**no results were obtained due to subject not wanting to be tested

Second, the chimpanzees' responses on each trial for the control and test condition separate are depicted in Figure S1. This figure adds information regarding chimpanzees' responses over time.

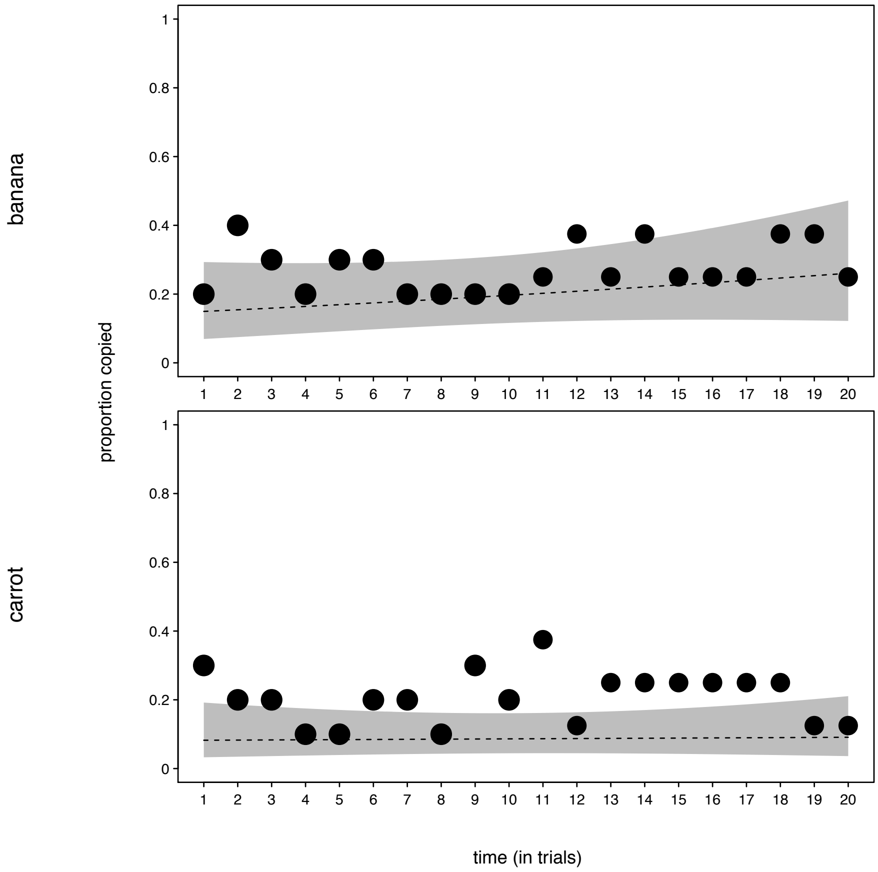

Figure S1. Proportion of demonstrator copying per trial for each condition (control condition = "carrot"; test condition = "banana"). The proportions are relatively low due to the fact that instead of copying the demonstrator, chimpanzees were mostly conservative and chose their trained token. Yet, when they copied the demonstrator, they did so more frequently when the demonstrator received banana (test condition) compared to when the demonstrator received carrot (control condition). The dotted line indicates the model estimation, the grey area the 95% confidence intervals.

Third, we present results from a derived GLMM in which subjects' choices for their trained token-type are omitted, such that their choice comprises a dichotomous one between copying the demonstrator or using the third, random option. This model is structured in the exact same way as the reported GLMM with response variable "yes/no copied the demonstrator", yet adds insight on the subjects' relative choosing between

copying the demonstrator and the third, random option across conditions in one model (n=143). The effect of "condition" also in this model is highly significant (χ^2 = 36.13, Δdf = 1, p < 0.001; Estimate ± SE = -10.84 ± 3.72), corroborating the findings reported in the main text.

Finally, we present the GLMM results for all estimated parameters in the copy-if-better model (response variable is "yes/no copied the demonstrator"). We present both the model summaries and the single term analyses based on model comparisons (comparing the deviance of the models with and without the concerning parameter). Note that in order to determine whether a factor significantly contributes to the prediction of subjects' responses, the single term analyses (based on Maximum Likelihood Ratio tests) results are conclusive [9].

Table S2a. Model summary for the copy-if-better model: Estimates, standard errors, *z*-values, and *p*-values obtained from the GLMM analysis (including all trials except for the first one for each subject per condition; n=340)

Coefficients	*Estimate*	*SE*	*z-value*	*p(>|z|)*
(Intercept)	-7.5259	2.6714	-2.817	0.004843**
Trained.token (green)	-0.2931	3.1437	-0.093	0.925714
Trained.token (grey)	-4.3545	3.2315	-1.348	0.177816
Demonstrator.token (green)	3.2906	1.3396	2.456	0.014031*
Demonstrator.token (grey)	6.7396	1.9220	3.507	0.000454***
Trial	-1.2971	0.6333	-2.048	0.040538*
Condition (carrot)	-2.1303	0.8022	-2.656	0.007915**
Order	0.1740	2.5216	0.069	0.944979

*** p<0.001; ** p<0.01 * p<0.05

Table S2b. Single term analyses for the copy-if-better model: Degrees of Freedom, Likelihood Ratio Test statistic (χ^2) and *p*-values obtained from model comparisons (Maximum Likelihood Ratio tests).

Coefficients	*Df*	*LRT*	*p(Chi)*
Trained.token	2	1.9013	0.3864843
Demonstrator.token	2	18.1575	0.0001141***
Trial	1	4.3198	0.0376706*
Condition	1	9.1748	0.0024537**
Order	1	0.0047	0.9453866

*** p<0.001; ** p<0.01 * p<0.05

Interpretation of control variables

In our experimental setup and in our GLMM analyses, we controlled for the effects of the specific token-type the subject was trained on (trained.token), the specific token-type used by the demonstrator (demonstrator.token) and the order by which subjects participated in the test and control conditions by counterbalancing all relevant factors and incorporating them in our GLMM models as fixed effects, respectively. We found no significant effects of order and trained.token, and a significant effect of demonstrator.token (see Table S2b). Inspecting the model summaries, we find that whenever the demonstrator was using the green or grey token, she was more likely to be copied compared to when she was using the blue token (see Table S2a). Additionally, we report that chimpanzees seemed decreasingly inclined to copy the demonstrator over time across conditions (main effect for trial; see Table S2a). Lastly, in line with the results depicted in Table S1, we found significant differences between the chimpanzees in their proclivity to apply a copy-if-better strategy across the entire set of trials, for which we controlled in the random effect structure of our GLMM (subject: $\chi^2 = 40.2$, $\Delta df = 1$, $p < 0.001$). We chose not to report the results of these control variables in the

main text, however, because the results relevant to our hypotheses (the effect of "condition") exist despite their respective influences (also see L129-132 in main text).

DISCUSSION

Note that our paradigm simultaneously represents an inequity aversion design with the additional feature of offering the disadvantaged individual a way out of his predicament. By rewarding the demonstrator with a higher value reward than the subject, while both individuals engage in a similar token-exchange procedure (i.e. there is no difference in the amount of required effort between the individuals), we created an inequity between the two individuals that is typically used in inequity aversion studies (e.g. Bräuer et al. 2009; Brosnan et al. 2010). In contrast to these studies, however, our paradigm incorporated an option for the disadvantaged individual to undo the inequity and therefore level the playing field (also see Hopper et al. 2013). We envisage that offering individuals alternatives to the inequity inflicted upon them will yield interesting insights into their (lack of) flexible application of (social) strategies.

REFERENCES

1. Sánchez-Amaro A., Pereto A., Call J. 2015 Differences in between-reinforcer value modulate the selective-value effect in great apes (Pan troglodyes, P. paniscus, Gorilla gorilla, Pongo abelii). *Journal of Comparative Psychology*. (doi:10.1037/com0000014).
2. Hrubesch C., Preuschoft S., van Schaik C. 2009 Skill mastery inhibits adoption of observed alternative solutions among chimpanzees (Pan troglodytes). *Animal Cognition* **12**(2), 209-216. (doi:10.1007/s10071-008-0183-y).
3. Marshall-Pescini S., Whiten A. 2008 Chimpanzees (Pan troglodytes) and the question of cumulative culture: an experimental approach. *Animal Cognition* **11**(3), 449-456. (doi:10.1007/s10071-007-0135-y).
4. van Leeuwen E.J.C., Cronin K.A., Schütte S., Call J., Haun D.B.M. 2013 Chimpanzees flexibly adjust their behaviour in order to maximize payoffs, not to conform to majorities. *Plos One* **8**(11). (doi:10.1371/journal.pone.0080945).
5. Yamamoto S., Humle T., Tanaka M. 2013 Basis for cumulative cultural evolution in chimpanzees: social learning of a more efficient tool-use technique. *Plos One* **8**(1). (doi:e55768).
6. R Core Team. 2015 R: A language and environment for statistical computing. R Foundation for Statistical Computing, Vienna, Austria. ISBN 3-900051-07-0, URL http://www.R-project.org/
7. Bates, D., Maechler, M., Bolker, B., Walker, S. 2015 Fitting Linear Mixed-Effects Models Using lme4. *Journal of Statistical Software*, 67(1), 1-48. (doi:10.18637/jss.v067.i01).
8. Dobson A.J. 2002 An Introduction to Generalized Linear Models. Chapman & Hall/CRC. Boca Raton.
9. Bolker B.M, Brooks M.E, Clark C.J, Geange S.W, Poulsen J.R, Stevens M.H.H., White JS.S. 2008 Generalized linear mixed models: a practical guide for ecology and evolution. *Trends Ecol Evol*, 24, 127-135.
10. Bräuer J, Call J, Tomasello M. 2009. Are apes inequity averse? New data on the token-exchange paradigm. American Journal of Primatology 71:175-181
11. Brosnan SF, Talbot C, Ahlgren M, Lambeth SP, Schapiro SJ. 2010. Mechanisms underlying responses to inequitable outcomes in chimpanzees, Pan troglodytes. Animal Behaviour 79:1229-1237

12. Hopper LM, Lambeth SP, Schapiro SJ, Brosnan SF. (2013) When given the opportunity, chimpanzees maximize personal gain rather than "level the playing field". PeerJ 1:e165 https://doi.org/10.7717/peerj.165
