# Supplementary material for: Conservatism and “copy-if-better” in chimpanzees (Pan troglodytes)
Source: Anim Cogn. 2016 Dec 20;20(3):575–9. doi: 10.1007/s10071-016-1061-7 (PMC5394136; doi:10.1007/s10071-016-1061-7)
Supplement: Supplementary file 2 — Supplementary material 2 (PDF 40 kb) [file 10071_2016_1061_MOESM2_ESM.pdf]

```
#Conservatism & Copy if better in Chimpanzees (WKPRC)

rm(list=ls())
setwd("~/Desktop/Copy if better")
xdata=read.table(file="Copy if better.txt", header=T, sep="\t")
str(xdata)
head(xdata)
nrow(xdata)
table(xdata$condition)
library(lme4)
contr=glmerControl(optimizer="bobyqa",optCtrl=list(maxfun=100000000))
xdata$order=as.factor(xdata$order)
xdata$z.trial=scale(xdata$trial)

#xdata=xdata[xdata$trial!="1",]
#xdata=xdata[xdata$trial=="2" |xdata$trial=="12",]
#xdata=xdata[xdata$trained!="1",]

#GLMM (binomial error structure, yes/no used trained token-type = "trained")

res=glmer(trained~trained.token+stooge.token+z.trial*condition+order
 +offset(log(demonstrations.observed))+(1|subject)+(1|test.date)+(0+z.trial|
 subject), family=binomial, data=xdata, control=contr)

red=glmer(trained~trained.token+stooge.token+order
 +offset(log(demonstrations.observed))+(1|subject)+(1|test.date)+(0+z.trial|
 subject), family=binomial, data=xdata, control=contr)

#xdata$condition=relevel(xdata$condition, ref="carrot")

anova(red, res, test="chisq")
summary(res)
drop1(res, test="Chisq")

#GLMM (binomial error structure, yes/no copied demonstrator = "observed")

res=glmer(observed~trained.token+stooge.token+z.trial*condition+order
 +offset(log(demonstrations.observed))+(1|subject)+(1|test.date)+(0+z.trial|
 subject), family=binomial, data=xdata, control=contr)

red=glmer(observed~trained.token+stooge.token+order
 +offset(log(demonstrations.observed))+(1|subject)+(1|test.date)+(0+z.trial|
 subject), family=binomial, data=xdata, control=contr)

res1=glmer(observed~trained.token+stooge.token+z.trial+condition+order
 +offset(log(demonstrations.observed))+(1|subject)+(1|test.date)+(0+z.trial|
 subject), family=binomial, data=xdata, control=contr)

xdata$condition=relevel(xdata$condition, ref="carrot")

anova(red, res, test="chisq")
```

```
anova(res1, res, test="chisq")
summary(res)
summary(res1)
drop1(res, test="Chisq")
drop1(res1, test="Chisq")

#means and sems for both conditions, change "carrot" into "banana"

xdata=xdata[xdata$condition=="carrot",]
nrow(xdata)

x1=tapply(xdata$trained, xdata$trial, mean)
x1=matrix(x1)
x1=na.omit(x1)
mean(x1)
nrow(x1)
sd(x1)
xx=sd(x1)
sem=(xx/sqrt(length(x1)))
sem

x1=tapply(xdata$observed, xdata$trial, mean)
x1=matrix(x1)
x1=na.omit(x1)
mean(x1)
nrow(x1)
sd(x1)
xx=sd(x1)
sem=(xx/sqrt(length(x1)))
sem

x1=tapply(xdata$random, xdata$trial, mean)
x1=matrix(x1)
x1=na.omit(x1)
mean(x1)
nrow(x1)
sd(x1)
xx=sd(x1)
sem=(xx/sqrt(length(x1)))
sem

#plot bar graphs means ± sems

means.overall=c(0.5825, 0.5775, 0.2075, 0.275, 0.21, 0.1475)
sem.overall=c(0.01987626, 0.01875219, 0.01711993, 0.01581139, 0.01510925,
 0.01952562)

#pdf(file="Token choices (trials)_other legend.pdf", width=7, height=7)

choices=barplot(t(matrix(means.overall, ncol=2, byrow=TRUE,
 dimnames=list(c("Trained token", "Observed token", "Random token"),
```

```
c("Control condition", "Test condition")))), ylim=c(0,1), col=c("black",
"gray"), beside=TRUE, legend.text=TRUE, args.legend=list(x="topright"),
ylab="Token choices (in proportion)")

#axis(1, labels=FALSE, at=friendship)
#axis(2, at=seq(0,1, by=0.2))
#main="XX"

segments(choices, means.overall-sem.overall, choices, means.overall
+sem.overall, lwd=1)
segments(choices - 0.1, means.overall-sem.overall, choices+0.1, means.overall-
sem.overall, lwd=1)
segments(choices - 0.1, means.overall+sem.overall, choices+0.1, means.overall
+sem.overall, lwd=1)

#adding significance asterisks
#points(7.5, 0.85, pch="***")

#dev.off()

#model stability removing one observation at the time
stability.results<-c()
for(i in 1:nrow(xdata)){
    stability.model<-glmer(observed~trained.token+stooge.token+z.trial
+condition+order+(1|subject)+(1|test.date)+(0+z.trial|subject),
family=binomial, data=xdata[-i,], control=contr)

stability.results<-rbind(stability.results, coefficients(stability.model))
}

stability.results=as.data.frame(stability.results)

yy=c()

for(i in 1:length(stability.results$subject)){

xx=stability.results$subject[i]
xx=as.data.frame(xx)

yy=rbind(yy, c(xx$trained.tokengreen[1],xx$trained.tokengrey[1],xx
$stooge.tokengreen[1],xx$stooge.tokengrey[1],xx$z.trial[1],xx
$conditioncarrot[1],xx$order[1],xx$z.trial.conditioncarrot[1]))

}

#plot interaction trial X condition (for ESM)

source("draw.2.w.int.bw.1.cov.and.1.fac.r")

# xaxt.labels=4:14
# xaxt.at=(xaxt.labels-mean(xdata$age))/sd(xdata$age)
```

```
# yaxt.at=seq(0, 1, by=0.2)
# yaxt.labels=as.character(yaxt.at)
# yaxt.labels[nchar(yaxt.labels)==1]=paste(yaxt.labels[nchar(yaxt.labels)==1],
 "0", sep=".")

str(xdata)

xdata$z.trial=as.vector(scale(xdata$trial))

trained.code1=as.numeric(xdata$trained.token==levels(xdata$trained.token)[2])
trained.code2=as.numeric(xdata$trained.token==levels(xdata$trained.token)[3])
trained.code1=trained.code1-mean(trained.code1)
trained.code2=trained.code2-mean(trained.code2)

stooge.code1=as.numeric(xdata$stooge.token==levels(xdata$stooge.token)[2])
stooge.code2=as.numeric(xdata$stooge.token==levels(xdata$stooge.token)[3])
stooge.code1=stooge.code1-mean(stooge.code1)
stooge.code2=stooge.code2-mean(stooge.code2)

condition.code=as.numeric(xdata$condition==levels(xdata$condition)[2])
condition.code=condition.code-mean(condition.code)

order.code=as.numeric(xdata$order==levels(xdata$order)[2])
order.code=order.code-mean(order.code)

plot.model=glm(observed~trained.code1+trained.code2+stooge.code1+stooge.code2+
 z.trial*condition+order.code, family=binomial, data=xdata)

source("ci_glm.r")
cis=ci.glm(model.res=plot.model, resol=100, level=0.95, use=c("condition",
 "z.trial"))

xaxt.labels=1:20
xaxt.at=(xaxt.labels-mean(xdata$trial))/sd(xdata$trial)
yaxt.at=seq(0, 1, by=0.2)
yaxt.labels=as.character(yaxt.at)
yaxt.labels[nchar(yaxt.labels)==1]=paste(yaxt.labels[nchar(yaxt.labels)==1],
 "0", sep=".")

#pdf(file="Copy probability across trials.pdf", width=7, height=7)

draw.2.w.int.bw.1.cov.and.1.fac(
  plot.data=xdata, covariate="z.trial", factor="condition", response=xdata
 $observed, coefs=coefficients(plot.model),
    link=c("logit"),
  grid.resol=NA, xaxt.at=xaxt.at, xaxt.labels=xaxt.labels, yaxt.labels=NULL,
 yaxt.at=NULL, xlim=NULL, ylim=NULL, xlab="time (in trials)", ylab="proportion
 copied", size.fac=1,
    add.q=F, e.type="T", conf.int=cis, ci.type=c("area"), fac.level.seq=NULL,
 fac.level.labels=NULL,
    all.in.one=F, ltys=NULL, pchs=NULL, lwd=1, pcols=NULL,
```

```
legend.where="topright", legend.what=NULL, add.lines=F, reset.par=T, quiet=T,
besides=F)

#dev.off()
```
